# Apolipoprotein E gene polymorphism and risk of type 2 diabetes and cardiovascular disease

**DOI:** 10.1186/s12933-016-0329-1

**Published:** 2016-01-22

**Authors:** Dalia El-Lebedy, Hala M. Raslan, Asmaa M. Mohammed

**Affiliations:** Medical Research Division, Department of Clinical and Chemical Pathology, National Research Centre, Al-Bohouth Street, Cairo, 12311 Egypt; Medical Research Division, Department of Internal Medicine, National Research Centre, Cairo, Egypt; Department of Environmental and Occupational Medicine, National Research Centre, Cairo, Egypt

**Keywords:** Apolipoprotein E, Polymorphism, Type 2 diabetes mellitus, Cardiovascular disease

## Abstract

**Background:**

Lipoprotein-related mechanisms have been associated with damage to the cardiovascular system in diabetic patients. Apolipoprotein E gene which affects the clearance of lipoproteins and consequently the lipid profile in our body is one of the most studied candidate genes and recently has been reported to be associated with T2DM and CAD. In this work, we studied the association of apoE gene polymorphism with T2DM and CVD and its effect on plasma lipids profile.

**Methods:**

Our study was conducted on 284 subjects categorized into 100 patients with T2DM, 100 patients with T2DM complicated with CVD and 84 normal control subjects. ApoE gene polymorphism was genotyped by real-time PCR using TaqMan^®^ SNP Genotyping Assay.

**Results:**

ApoE E3/E3 genotype was the most common in our subjects. The frequencies of E3/E4 genotype and ε4 allele were increased in both T2DM patients and CVD patients as compared with controls, but were significant only in CVD patients (p = 0.004 and 0.007, respectively). Diabetic patients who carried E3/E4 genotype were at 2.4-fold increased risk to develop CVD (95 % CI 1.14–5.19, P = 0.02) and the ε4 allele associated with 2.23-fold higher CVD risk (95 % CI 1.09–4.59, P = 0.02). After adjustment for other established risk factors, E3/E4 genotype was an independent risk factor for CVD (OR = 2.3, p = 0.009) but not for T2DM (OR = 1.7, p = 0.28), while ε4 allele was an independent risk factor for both T2DM (OR = 2.2, p = 0.04) and CVD (OR = 3.0, p = 0.018) with 5.9-fold increased risk to develop CVD in T2DM patients (p = 0.019). E3/E4 genotype associated with significantly higher levels of TC and non HDL-C in all groups and with significantly higher levels of LDL-C in both T2DM and CVD patients.

**Conclusions:**

ApoE gene polymorphisms associate with CVD and affect the lipid profile. The ε4 allele is an independent risk factor for both T2DM and CVD. Further genetic studies to add information beyond the traditional cardiovascular risk factors in T2DM and to identify risk genotypes will help in early prediction and identification of at risk patients.

## Background

Type 2 diabetes mellitus (T2DM) is one of the most common diseases with a high incidence and prevalence throughout the world [1]. The prevalence of type 2 diabetes is rising at an alarming rate due to increase in life expectancy, obesity, physical inactivity and adoption of sedentary lifestyles [2]. In 2013, the International Diabetes Federation (IDF) estimated that almost 366 million people (8.3 % of the adult population) around the world have diabetes, and 280 million people (6.4 % of the adult population) have impaired glucose tolerance (IGT), a major risk factor for type 2 diabetes. By 2030, these figures are expected to rise to 552 million (9.9 % of the world’s population) and 398 million (7.1 % of the adult population), respectively. In developing countries, the number of people with diabetes will increase by 150 % in the next 25 years [3]. Egypt is in the world 8^th^ place in terms of diabetes incidence, affecting up to 9.3 % of population, and due to a rapidly increasing and ageing population, Egypt will have the highest projected number of people with diabetes in the region by 2025 [3].

T2DM is a major independent risk factor for cardiovascular disease (CVD), the most common cause of morbidity and mortality among diabetic patients [4]. The increasing incidence of non-communicable diseases (NCDs) including diabetes and cardiovascular diseases, places a huge burden on Egypt’s healthcare resources and about 41 % of all deaths in Egypt are from NCDs [5]. Therefore, better understanding of the pathogenesis of CVD and T2DM is of great interest for early prediction and identification of at-risk patients and for better clinical management.

Modifiable factors such as dyslipidemia, obesity, oxidative stress, smoking, exercise and alcohol intake, as well as non-modifiable factors: age, sex, positive family history and genetic predisposition of the individual, have been identified as risk factors for both T2DM and CVD [6].

Lipoprotein- related mechanisms have been associated with damage to the cardiovascular system in diabetic patients [7]. Apolipoprotein E (ApoE) gene which affects the clearance of lipoproteins [8] and, consequently, the lipid profile in our body [9] is one of the most studied candidate genes for T2DM and/or CVD in the last decade.

ApoE gene, located on the long arm of chromosome 19 at position q13.2, is a polymorphic gene with single nucleotide polymorphisms (SNPs) at positions 112 and 158 resulting in three major alleles: ε2, ε3, and ε4, coding for 3 isoforms: apoE2 (Cys112/Cys158), the most common apoE3 (Cys112/Arg158) and apoE4 (Arg112/Arg158) with 6 possible genotypes: E2/E2, E2/E3, E3/E3, E3/E4, E4/E4 and E2/E4 [6, 10].

ApoE isoforms have different effects on the metabolism of lipoproteins. Allele ε2 is associated with lower plasma levels of LDL cholesterol and lower risk of coronary artery disease (CAD) [11], meanwhile, ε4 allele is associated with higher plasma levels of total cholesterol (TC), LDL-C, very low-density lipoprotein cholesterol (VLDL-C), and greater risk of CAD when compared with ε3 allele [12]. Impaired lipid clearance by apoE ε4 is attributed to its higher affinity to LDL-R compared to other alleles leading to early “receptor occupation” and accumulation of LDL particles which suppress LDL-R synthesis resulting in lower clearance of lipoproteins from the body through LDL-R [13]. Also, a direct relationship between apoE isoforms and premature atherosclerosis has been reported [6] and recently apoE ε4 allele has been associated with the development of both T2DM and CAD [14]. The aim of this study is to investigate the association of apoE gene polymorphism with T2DM and CVD and its effect on plasma lipid levels.

## Methods

### Subjects

Studied subjects were recruited from the Outpatients Clinic of the National Research Centre and the National Diabetes and Endocrinology Institute. Data of family and medical history, smoking habits and physical activity was obtained by questionnaire. Physical activity was defined as exercise for 2–3 days/week for at least 30 min. Clinical examination including measurement of systolic blood pressure (SBP) and diastolic blood pressure (DBP) was applied. Anthropometric measurements (weight and height) were collected and used for BMI calculation according to the standard formula BMI = weight (kg)/[height (m)]^2^. Hypertension was defined as blood pressure above 140/90 mmHg or taking antihypertensive drugs. Dyslipidemia was defined as level of total cholesterol (TC) >200 mg/dL, triglycerides (TG) >150 mg/dL, LDL-C >130 mg/dL, HDL-C <40 mg/dL, TC/HDL-C ratio >4.0 or under medication of lipid lowering drugs [15]. According to the criteria of American Diabetes Association [16], studied subjects were classified into 3 groups:

*Control group* included 84 healthy subjects with fasting plasma glucose (FPG) <100 mg/dL. Exclusion criteria were hyperlipidemia, hypertension, CVD or family history of any form of CVD, diabetes mellitus, hepatic and renal diseases, endocrine disease, metabolic disorders, autoimmune diseases and those under medication.

*T2DM patients without CVD* included 100 subjects fulfilled the diabetes mellitus diagnostic criteria of FPG ≥126 mg/dL or under diabetes medication (oral and/or insulin) with no history or signs of any CVD. Exclusion criteria included renal disease, hepatic disease, endocrine disease, metabolic disorders and autoimmune diseases.

*T2DM complicated with CVD* included 100 subjects diagnosed to have diabetes with FPG ≥126 mg/dL or under diabetes medication and complicated with any of the vascular disease e.g. ischemic heart disease (IHD), macroangiopathy and/or cerebrovascular disease. Exclusion criteria included renal disease, hepatic disease, endocrine disease, metabolic disorders and autoimmune diseases.

Informed consent was obtained from all subjects and the study protocol was approved by the Ethics Committee of the National Research Centre.

### Lipid analysis and biochemical markers

Venous blood samples were collected from all subjects after 12 h fast. Total cholesterol (TC), Triglycerides (TG), high density lipoprotein cholestrol (HDL-C), low density lipoprotein cholestrol (LDL-C), fasting plasma glucose (FPG) were assayed on Roche Diagnostics clinical chemistry auto analyzer c311 (Germany). Glycosylated hemoglobin (HbA1c) was measured by high-performance liquid chromatographic (HPLC) method using Agilent 1100 series HPLC system (Agilent Technologies, Germany). VLDL-C level was calculated using the following equation: VLDL-C = (TC − LDL-C − HDL-C). Non-HDL-C level was calculated by subtracting HDL-C value from TC value [17].

### ApoE genotyping

Genomic DNA was extracted from 2 ml of whole peripheral blood using QIAamp DNA extraction kit (Qiagen Hilden, Germany, Cat no. 51304) according to the manufacturer’s protocol.

ApoE gene was genotyped using TaqMan^®^ SNP Genotyping Assays. SNPs at positions 112 (rs429358) and 158 (rs7412) determined the encoded alleles, ε2 (rs429358-T + rs7412-T), ε4 (rs429358-C + rs7412-C) and ε3 allele (rs429358-T + rs7412-C). All primers and probes were designed by Applied Biosystems (Foster City, CA, USA) and genotyping analyses were performed on ABI 7500 Real Time PCR system (Applied Biosystems) according to the manufacturer’s protocol. For genotyping quality control, negative controls were included in all SNPs and 10 % of samples were randomly selected and analyzed in duplicates and the concordance rate was 100 %.

### Statistical analysis

The collected data and the clinical results have been statistically analyzed using IBM SPSS version 20.0 software (Statistical Package for Social Science). Quantitative data were expressed as mean values ± standard deviation (SD). Ranges and frequency of distributions were estimated for quantitative variables. Normally distributed data were compared using Student’s *t* test for 2 groups and ANOVA test for more than 2 groups. The significance of differences between proportions was tested by the Chi square test (χ^2^). Differences were considered significant with p value <0.05. Allele and genotype differences between groups and deviations from Hardy–Weinberg equilibrium were tested by Chi square test. Univariable logistic regression analysis was used to test the association between diseases and Apo E gene polymorphism and presented as unadjusted odds ratios (OR) with confidence interval (95 % CI). Multivariate logistic regression analysis was used to determine the risk factors for development of diseases with adjustment for potential covariates: age, gender, BMI, smoking status and physical activity and presented as adjusted ORs.

## Results

### General characteristics and biochemical variables of the study population

The study included 284 subjects classified into 100 patients with T2DM, 100 patients with T2DM + CVD, and 84 control subjects. Their age ranged from 40 to 68 years. The frequencies of CVDs in our patients were: 67 % ischemic heart disease (IHD), 13 % cerebrovascular disease, 11 % macroangiopathy, 6 % combined IHD and cerebrovascular disease, and 3 % combined macroangiopathy and cerebrovascular disease.

A significant age difference was found between control subjects and CVD patients (p < 0.0001) implying a higher risk of developing CVD with increasing age. Also a significant sex difference was found between CVD patients and, both, controls (p = 0.003) and T2DM patients (p = 0.01), signifying that male gender is associated with higher chances to develop CVD and T2DM. Diabetic patients with CVD had lower levels of physical activity (p = 0.005) and longer durations of diabetes (p = 0.001) when compared to controls and T2DM patients. BMI, SBP and DBP were significantly higher in patient groups than in controls. Higher frequencies of hypertension (p < 0.0001) and smoking (p = 0.01) were observed among CVD patients when compared to T2DM patients. Plasma glucose and HbA1C levels were significantly higher in patients compared to controls (p < 0.0001).

Lipid profile data showed a significant association between dyslipidemia and CVD in diabetic patients (p < 0.0001). Significantly higher levels of TC, TG, LDL-C, VLDL-C, non HDL-C, TC/HDL-C and lower levels of HDL-C were demonstrated in patients compared to controls. CVD patients showed significantly lower levels of HDL-C (p = 0.018) and higher levels of TC (p = 0.027), VLDL-C (p = 0.015), non HDL-C (p = 0.009) than T2DM patients. Demographic, clinical and biochemical data of enrolled subjects are summarized in Table 1.

**Table 1:** Demographic, clinical and biochemical data of the study population

Variable	Controls (n = 84)	T2DM (n = 100)	T2DM + CVD (n = 100)
Age (years)	51.8 ± 5.2	50.9 ± 7.5	58.3 ± 7.2**
Sex (male/female)	45/39	57/43	74/26**^†^
BMI (kg/m^2^)	23.21 ± 4.62	27.59 ± 4.90*	29.81 ± 5.55**
SBP (mmHg)	115.3 ± 9.6	131.5 ± 19.7*	145.1 ± 22.6**†
DBP (mmHg)	77.6 ± 6.9	79.7 ± 18.0*	90.6 ± 9.7**†
Hypertension (%)	–	31.4	94.7^†^
Smokers (%)	11.1	7.8	15.7^†^
Physical activity (%)	66.7	54.9	31.6**^†^
Diabetes duration (years)	–	8.4 ± 7.2	13.3 ± 7.4^†^ 13.00 ± 6.97
Dyslipidemia (%)	–	72.5	92.1^†^
Glucose (mg/dL)	85 ± 9.5	147.1 ± 64.1*	161 ± 66.2**
HbA1c (%)	5.3 ± 0.6	6.4 ± 1.2*	6.6 ± 1.3**
Triglyceride (mg/dL)	118.3 ± 31.1	145.6 ± 72*	164.5 ± 66.2**
TC (mg/dL)	175.7 ± 16	192.1 ± 48.4*	212.7 ± 47.9**^†^
LDL-C (mg/dL)	104.7 ± 7	118.22 ± 38.9*	123 ± 43.7**
HDL-C (mg/dL)	53.9 ± 9.7	49.2 ± 11.2*	43.9 ± 11.8**^†^
VLDL-C (mg/dL)	17.1 ± 24.2	29.1 ± 14.4*	39.6 ± 22.9**^†^
Non-HDL-C (mg/dL)	121.14 ± 15.9	147.8 ± 44.9*	162.7 ± 47.7*^†^
TC/HDL-C	3.3 ± 0.6	4.4 ± 1.2*	4.5 ± 1.2**

*BMI* body mass index, *SBP* systolic blood pressure, *DBP* diastolic blood pressure, *HbA1c* hemoglobin A1C, *TC* total cholesterol, *LDL-C* low density lipoprotein cholesterol, *HDL-C* high density lipoprotein cholesterol, *VLDL-C* very low density lipoprotein cholesterol

* Significant p in comparison between controls and T2DM

** Significant p in comparison between controls and T2DM + CVD

^†^Significant p in comparison between T2DM and T2DM + CVD

### ApoE genotype and allele frequencies in patients and controls

**Fig. 1:**
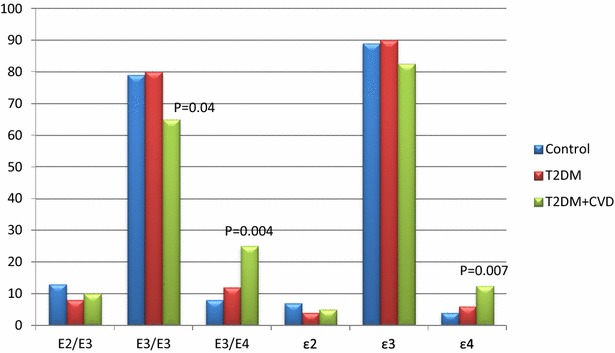
Genotypes distribution and alleles’ frequency of ApoE gene polymorphism in patients and controls

The genotype distributions of all groups were in Hardy–Weinberg equilibrium (p > 0.05). E3/E3 was the most common genotype in our subjects. Genotype and allele frequencies among studied groups showed significant differences (p = 0.014 and 0.023, respectively). In CVD patients, the frequency of E3/E3 genotype was significantly lower (p = 0.04), while the frequencies of E3/E4 genotype and ε4 allele were significantly higher when compared to controls (p = 0.004 and 0.007, respectively). In T2DM patients, though E3/E4 genotype and ε4 allele frequencies were higher than in controls, the differences were not significant (p = 0.41 and 0.43, respectively) (Fig. 1).

### Association of apoE gene polymorphism with T2DM and CVD

**Fig. 2:**
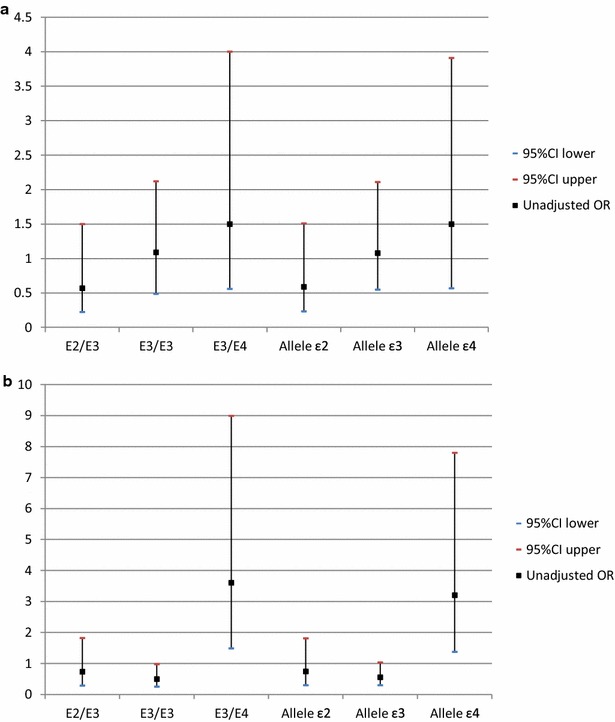
Association of ApoE gene polymorphism with the risk of T2DM (**a**), CVD (**b**)

Univariate analysis to study the association of different genotypes and alleles with the risk of T2DM and CVD as compared to healthy controls showed that E3/E4 genotype associated with 3.6-fold increased risk to develop CVD (P = 0.004) and ε4 allele associated with 3.2-fold increased CVD risk (p = 0.007). Although, E3/E4 genotype increased the risk for T2DM by 1.5 times, it was of no statistical significance (p = 0.41) (Fig. 2).

**Fig. 3:**
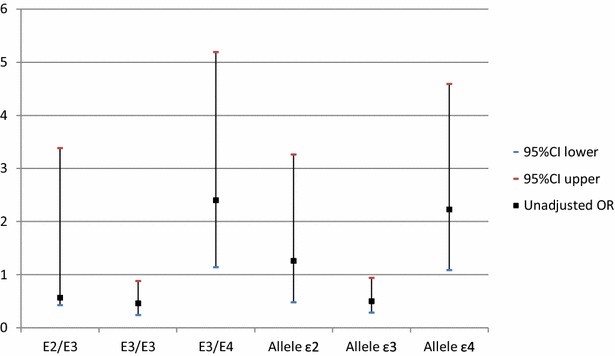
Associations of apoE gene polymorphism with the risk of CVD in T2DM patients

Association studies for the CVD risk in T2DM patients revealed that diabetic patient who carried E3/E4 genotype were at 2.4-fold increased risk to develop CVD (P = 0.02), while ε4 allele carriers were at 2.23-fold increased risk (P = 0.02) (Fig. 3).

**Fig. 4:**
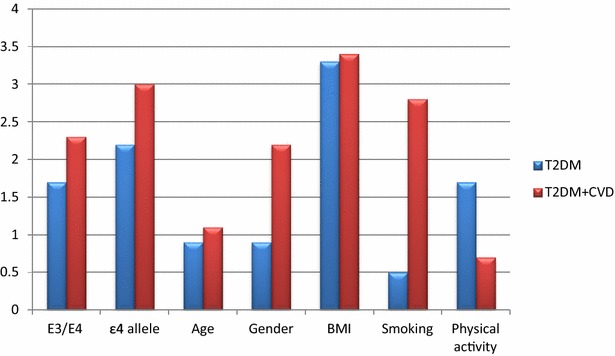
Adjusted odds ratio (OR) of the multivariate logistic regression analysis for T2DM and CVD risk

**Fig. 5:**
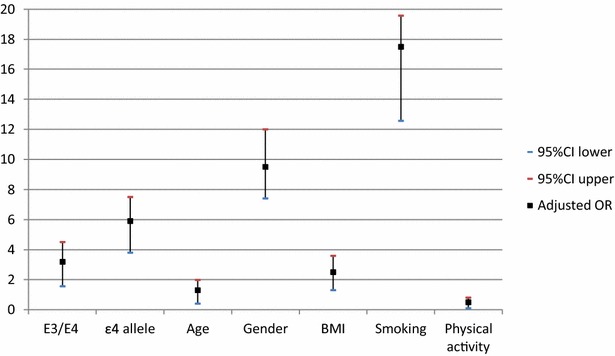
Adjusted odds ratio (OR) of the multivariate logistic regression analysis for CVD risk in T2DM patients

Multivariate logistic regression analysis after adjustment for other established risk factors: age, male gender, BMI, smoking status and physical activity, showed that E3/E4 genotype was an independent risk factor for CVD (p = 0.009) but not for T2DM (p = 0.28). Allele ε4 was an independent risk factor for both T2DM (p = 0.04) and CVD (p = 0.018) and increased the risk of CVD in T2DM patients by 5.9 folds (p = 0.019). As regards other covariates, age, BMI, male gender and physical activity were independent risk factors for CVD, while BMI was the only independent risk factor for T2DM (Fig. 4). For CVD risk in T2DM patients, age, male gender, BMI, smoking status and physical activity were the independent risk factors. Diabetic smokers were 17.5 times more-at risk to develop CVD than non- smokers (p = 0.013) (Fig. 5).

### ApoE genotypes and plasma lipids profile

By comparing the plasma lipid profile parameters in the E4-bearing genotype in our study i.e. E3/E4 with E3/E3 genotype as a reference, we found that E3/E4 genotype associated with significantly higher levels of TC and non HDL-C in all groups and significantly higher levels of LDL-C in both T2DM and CVD patients. Though, E3/E4 genotype carriers have higher levels of VLDL-C and lower levels of HDL-C, yet the differences were not significant (Table 2).

**Table 2:** Lipid profile parameters in apoE E3/E3 vs. E3/E4 genotypes

Parameter (mg/dl)	Control		T2DM		T2DM + CVD	
	E3/E3 (n = 66)	E3/E4 (n = 7)	E3/E3 (n = 80)	E3/E4 (n = 12)	E3/E3 (n = 65)	E3/E4 (n = 25)
TC	171.24 ± 25.15	189.42 ± 8.52**	191.47 ± 51.06	223.30 ± 22.80**	204.55 ± 42.35	221.56 ± 70.37*
TG	114.99 ± 32.08	121.71 ± 31.19	153.78 ± 74.98	145.92 ± 65.05	159.72 ± 61.47	149.08 ± 79.28
HDL-C	53.14 ± 10.97	50.85 ± 9.24	44 ± 12.52	42.38 ± 11.08	50.52 ± 10.85	48.86 ± 13.49
LDL-C	109.06 ± 20.40	96.28 ± 4.85	116.72 ± 40.17	151.76 ± 18.85**	121.67 ± 43.16	135 ± 61.85*
VLDL-C	20 ± 9.6	21 ± 11.8	29.75 ± 14.95	31.15 ± 12.61	32.35 ± 12.16	35.7 ± 23.15
Non-HDL-C	118 ± 19.4	139 ± 13**	147.47 ± 46.27	180.92 ± 23.76**	154.03 ± 41.29	170.7 ± 61.77*

Data presented as mean ± SD

* p value < 0.05

** p value < 0.01

## Discussion

ApoE is a 299-amino acids plasma glycoprotein which acts as a high affinity ligand for several hepatic lipoprotein receptors such as LDL-R and LDL-related protein (LRP1) and is involved in several lipoproteins metabolism, transport and digestion [18]. Dyslipidemia or dyslipoproteinemia might strongly contribute in aggravating the micro- and macro-vascular complications and the related accelerated atherosclerosis in diabetic patients [7] and recently has been suggested to be associated with both T2DM and CAD [14]. Since, different apoE isoforms associate with significant variation in lipid profiles [19], the coding apoE gene is a candidate for CVD and/or diabetes. In this work, we studied the association of apoE gene polymorphism with T2DM and CVD and its effect on plasma lipid parameters.

### ApoE gene polymorphism and risk of diabetes and CVD

To our knowledge this is the first study to investigate apoE gene polymorphism in T2DM with and without CVD in our population, E3/E3 was the most common genotype. Significant higher frequencies of E3/E4 genotype and ε4 allele were observed among our CVD patients. Individuals who carried E3/E4 genotype were at 3.6- fold higher risk to develop CVD while ε4 allele carriers were at 3.2-fold higher risk. In diabetic patients, E3/E4 genotype and ε4 allele increased the CVD risk by 2.4- and 2.23-folds, respectively. Multivariate regression analysis identified E3/E4 genotype as an independent risk factor for CVD, but not for T2DM, while ε4 allele was an independent risk factor for both T2DM and CVD and associated with 5.9-fold higher risk of CVD in T2DM patients.

Results from previous studies suggest that apoE ε4 allele has a variable significance in terms of predicting the risk of vascular events in different populations. In Finnish population, apoE genotypes were found to modulate the risk of coronary heart disease (CHD) and atherosclerotic vascular disease in non-insulin dependent diabetes mellitus (NIDDM) [20, 21]. Genotypes E4/E4 and E3/E4 associated with increased risk for CHD in NIDDM patients and the prevalence of CHD disease among diabetic patients with genotypes E4/E4 or E3/E4 was 81 vs. 58 % among patients with genotype E3/E3, and 53 % among those with genotypes E2/E2 or E3/E3 [20]. Also, E4-bearing genotypes associated with increased risk for macro and micro vascular complications in NIDDM patients both in men and women, in contrast to E2 phenotype which somehow protected from macroangiopathy and associated with lower plasma TC and LDL-C concentrations and lower plasma lipoprotein (a) levels [21]. In contrast, apo E polymorphism, notably, the ε4 allele was not found to influence the risk for cardiovascular disease in Italian diabetic patients and no significant differences among different genotypes were identified [22]. However, in another Italian study, apoE ε4 allele was reported as a risk factor for CAD and has been associated with low apoE concentrations [23]. In Greek patients with CAD, there was no significant association between ε4 allele and risk for CAD or myocardial infarction (MI), though a negative association of ε2 allele with Ml was observed [24]. Also, ε4 allele was not associated with an increased risk for CVD or ischemic vascular event (IVE) among Greek patients with CVD [25].

Meanwhile, significant association between ApoE polymorphism and CAD has been reported in several ethnic groups [12, 14, 26–34]. In Chinese study, apoE ε4 allele was reported to be associated with the increased risk of CAD in T2DM. Diabetic patients who carried E3/E4 or E4/E4 genotypes had higher concentrations of serum TC, LDL-C and lipoprotein (a) than patients with E2/E2 or E3/E2 genotypes, they also had the highest mortality rate (50 %) during 3–10 years follow-up period [12].

In Thai population, Apo ε4 allele has been reported as an independent risk factor for the development of both T2DM and CAD [14]. After adjusting for other risk factors, E3/E4 carriers showed 2.52-fold higher risk for CAD in T2DM patients. Moreover, allele ε4-bearing genotypes increased the risk of CAD by 2.32 folds and the risk of T2DM by 2.04 folds when compared to controls and the risk increased when combined with smoking and/or obesity confirming that development and progression of diabetes and CAD are multifactorial and no single factor can give a satisfactory explanation regarding the disease development [14].

In a recent meta-analysis evaluating the association of apoE gene polymorphism with atherosclerosis risk including subgroup analysis, the overall analysis and subgroup analysis based on ethnicities showed no significant association between apoE polymorphism and risk of atherosclerosis. However, subgroup analysis based on clinical phenotypes of atherosclerosis (clinical and subclinical atherosclerosis) showed that ε4 allele associated with incidence of clinical atherosclerosis [35].

In our study, age, BMI, male gender and physical activity were independent risk factors for CVD, while BMI was the only independent risk for T2DM. Diabetic smokers were 17.5 times more-at risk to develop CVD than non-smokers. Recently, smoking and obesity have been reported to be the two major risk factors that can promote the development of T2DM and CAD or both [14] which has been attributed to the enhanced oxidative stress that results in decreased insulin secretion and decreased uptake by the muscle cells [36]. Oxidative stress also increases vascular inflammation and involved in the development of CVD with or without combination of diabetes [37].

### Oxidative stress and vascular complications in diabetes

Hyperglycemia-induced oxidative stress in diabetic patients induces endothelial dysfunction which plays a central role in the pathogenesis of vascular diseases and may also increase pro-inflammatory and pro-coagulant factors expression [38]. Lower plasma levels of extracellular superoxide dismutase (EC-SOD), a major antioxidant enzyme, and higher plasma levels of advanced oxidation protein products (AOPP), markers of oxidative stress, have been detected and associated with myocardial infarction in diabetic patients [39].

Also, the anti-atherogenic properties of HDL might be affected by hyperglycemia. Glycated HDL has a reduced ability to protect against oxidation and in vitro studies showed that glycation can both inactivate PON1 (an enzyme that accounts for most of the antioxidant effect of HDL to prevent oxidation of LDL) and increase lipid peroxidation in HDL [40, 41]. Reduced PON-1 activity and concentration in studies of healthy subjects with elevated fasting glucose levels [42] and increased insulin resistance [43] support these in vitro data.

In a previous study on serum advanced glycation end products (AGEs) and its association with oxidative stress and PON-1 activity in T2DM patients, lower enzyme activity has been associated with vascular complications and AGEs showed a significant negative correlation with enzyme activity [44]. Apolipoprotein A-1 (apoA-I) is the major protein component of HDL that plays a major role in cholesterol homeostasis and exerts anti-inflammatory, antioxidant, and anti-atherogenic properties by stabilizing PON-1 enzyme [45]. In a recent study, glycated apoA-I has been reported to be associated with decreased activities of serum and HDL-associated PON1 and PON3 as well as the presence and severity of CAD in T2DM patients [46].

Recently, an interaction between the γ-glutamyltransferase-1 (GGT1) genotype and low serum levels of HDL-C has been identified in diabetic micro and macro angiopathy particularly among the G allele carriers, a variant that was associated with higher GGT serum levels. Increased pro-oxidant effect of GGT by increased LDL-associated GGT and GGT-mediated LDL-oxidation, together with coexisting low HDL-C, was suggested to be the mechanism linking GGT to the cardiovascular events [47].

### Effect of apoE genotype on plasma lipids

Our results showed that E3/E4 genotype associated with higher levels of TC and non HDL-C in all studied subjects, and with higher levels LDL-C in both T2DM and CVD patients highlighting the significance of increased LDL-C levels in T2DM and CVD development, in contrast to the previous report that non-HDL-C level is a strong predictor of CVD risk in T2DM and is particularly indicative of coronary events [48].

Results from previous studies regarding the relation between the apoE genotype and plasma lipids profile are inconsistent among different ethnic populations. In Indians, E3/E4 genotype was associated with lower HDL-C and higher LDL-C concentrations in CAD patients [49] and with higher TG levels in T2DM patients [50]. In Thai population, ε4 allele-diabetic carriers showed a significantly higher VLDL-C, TG and lower HDL-C levels compared to E3/E3 genotype carriers [14]. In a recent study on Kashmiri population, ε4 allele associated with significantly higher levels of LDL and TC in CAD patients [34]. ApoE ε4 allele associated with increased LDL-C in Tunisian men with T2DM [32] and with higher LDL-C and lower HDL-C levels in Spanish women with T2DM [51] suggesting that gender might affect the impact of apoE gene polymorphism on lipid parameters [14].

## Conclusion

Our study indicates that apoE gene polymorphisms associate with CVD and identifies apoE ε4 allele as an independent risk factor for both T2DM and CVD. Different apoE genotypes associate with significant variation in plasma lipids profile. Multiple risk factors interact and play a role in the development of T2DM and/or CVD. Further genetic studies to add information beyond the traditional cardiovascular risk factors in T2DM patients and to identify risk genotypes will help in early prediction and identification of at risk patients.

## Abbreviations

T2DM: type 2 diabetes mellitus
IDF: International Diabetes Federation
NCDs: non-communicable diseases
IGT: impaired glucose tolerance
CVD: cardiovascular disease
CAD: coronary artery disease
ApoE: apolipoprotein E
BMI: body-mass index
SBP: systolic blood pressure
DBP: diastolic blood pressure
FPG: fasting plasma glucose
TC: total cholesterol
TG: triglycerides
LDL: low-density lipoprotein
HDL: high-density lipoprotein
VLDL: very low density lipoprotein
HbA1c: glycosylated hemoglobin
CHD: coronary heart disease
IHD: ischemic heart disease
HPLC: high-performance liquid chromatographic
PCR: polymerase chain reaction
SNP: single nucleotide polymorphism
LDL-R: lipoprotein receptors
LRP1: LDL-related protein
CHD: coronary heart disease
NIDDM: non-insulin dependent diabetes mellitus
MI: myocardial infarction
IVE: ischemic vascular event
