# Erratum to: Apolipoprotein E gene polymorphism and risk of type 2 diabetes and cardiovascular disease

**DOI:** 10.1186/s12933-016-0354-0

**Published:** 2016-02-18

**Authors:** Dalia El-Lebedy, Hala M. Raslan, Asmaa M. Mohammed

**Affiliations:** Medical Research Division, Department of Clinical and Chemical Pathology, National Research Centre, Al‑Bohouth Street, Cairo, 12311 Egypt; Medical Research Division, Department of Internal Medicine, National Research Centre, Cairo, Egypt; Department of Environmental and Occupational Medicine, National Research Centre, Cairo, Egypt

## Erratum to: Cardiovasc Diabetol (2016) 15:12 DOI 10.1186/s12933-016-0329-1

After publication of the original article [1], the authors found that there was an error in Table 1. In Table 1 provided in the accepted submission, “13.00 ± 6.97” had mistakenly been included below the **Diabetes duration** row in the **T2DM** + **CVD** column.

The original article has been corrected in order to remove this unnecessary value from Table 1.
